# Pyriproxyfen treated surface exposure exhibits reproductive disruption in dengue vector *Aedes aegypti*

**DOI:** 10.1371/journal.pntd.0007842

**Published:** 2019-11-18

**Authors:** Kavita Yadav, Sunil Dhiman, BN Acharya, Rama Rao Ghorpade, Devanathan Sukumaran

**Affiliations:** 1 Vector Management Division, Defence Research and Development Establishment, Gwalior, Madhya Pradesh, India; 2 Synthetic Chemistry Division, Defence Research and Development Establishment, Gwalior, Madhya Pradesh, India; University of Queensland, AUSTRALIA

## Abstract

**Background:**

Reduced susceptibility of mosquito vectors to currently used insecticides hampers control interventions. Recently pyriproxyfen, an insect growth regulator has been demonstrated to effectively reduce the reproductive potential in vector mosquitoes.

**Methods:**

Pyriproxyfen (PPF), in different concentrations (0.75%, 0.075%, 0.0075%, and 0.00075%) was applied on papers and Indian wild type *Aedes aegypti* female mosquitoes (N ≥ 20 for each treatment) were exposed onto it as per WHO guidelines, to study the reproductive disruption. PPF concentration on treated papers was quantitatively cross-determined using HPLC method. Reduction in fecundity, fertility and adult emergence in exposed female *Ae*. *aegypti* was determined. Abnormal development in ovary and eggs of exposed females was studied microscopically after different time intervals.

**Results:**

Eggs laid, eggs hatched, pupae formed and adults emerged per female exposed in both before blood meal and after blood meal groups declined significantly from lowest to highest concentration of PPF (F ≥ 5.2; p < 0.02). Adult emergence inhibition in females exposed to PPF before and after blood meal groups ranged from 58.8% [OR = 0.18 (95% CI = 0.09–0.36)] to 79.2% [OR = 0.04 (95% CI = 0.02–0.10)] and 64.4% [OR = 0.12 (95% CI = 0.05–0.28)] to 77.1% [OR = 0.05 (95% CI = 0.02–0.14)] respectively in different concentrations. The probit model used suggested that FI_50_ (50% fertility inhibition) and EI_50_ (50% emergence inhibition) were 0.002% (p = 0.82) and 0.0001% (p = 0.99) for females exposed before blood meal, while 0.01% (p = 0.63) and <0.0001% (p = 0.98) for the females exposed after blood meal, respectively. The eggs laid by the females exposed to PPF-treated surface showed altered body organization, desegmentation and disoriented abdominal and cervical regions in the developing embryo. Quantification of PPF on impregnated papers showed that it was uniformly distributed throughout the matrix.

**Conclusions:**

The present study has shown that tarsal contact to PPF-treated surface for a small time drastically influenced the fecundity, fertility and adult emergence in Indian wild *Ae*. *aegypti* mosquitoes. Results suggest that a certain minimum concentration of PPF through contact exposure can reduce the abundance of vector mosquitoes to a considerable level. The formulations based on combination of PPF and other compatible insecticides may be an impactful approach where susceptible mosquitoes are killed by the insecticide component while resistant mosquitoes are sterilised by PPF.

## Author summary

Development of resistance against insecticides has challenged mosquito control programmes globally and prompted the research of alternative options that can complement insecticides. An insect growth regulator, pyriproxyfen (PPF) usage against mosquitoes can effectively reduce the vector population. PPF mainly inhibits the metamorphosis of mosquito larvae into pupae and prevent the adult emergence, therefore it is generally applied in mosquito breeding habitats. PPF has been shown to exhibit delayed residual effect that may impair the reproductive capacity by affecting the survival, fecundity and fertility of adult mosquito exposed through tarsal contact. Presently, the effectiveness of different concentrations of PPF intended to be delivered through contact have been evaluated against dengue vector *Ae*. *aegypti*. Results suggested that very low PPF concentration treated surfaces drastically reduce the fecundity, fertility and adult emergence in mosquitoes. Study suggests that control interventions based on PPF-treated surfaces could provide an additional route to target mosquito vector control by overall population density reduction.

## Introduction

Synthetic pyrethroids are currently WHO recommended and widely preferred insecticides in mosquito control due to their effectiveness and strong excito-repellency against various insect vectors. Primarily, the use of Long Lasting Insecticidal Nets (LLINs), Indoor Residual Spray (IRS) and other similar measures have substantially reduced many mosquito borne diseases in the endemic countries [1, 2]. However, the reduced mosquito susceptibility to recommended insecticide groups in different endemic regions may seriously deter the impact of control interventions using these insecticides [3, 4]. Additionally, the change in vectors biting pattern where mosquitoes adapt themselves to bite at the time when host is not using LLIN and sometime shifting their resting places to areas where IRS is not done, has further raised the concern for effective interventions [5, 6].

*Ae*. *aegypti* is an important vector of dengue and known to spread majority of dengue fever cases annually. Reports suggest that, although dengue fever is one of the major concerns in entire WHO region, but about 75% of the population at risk of dengue infection lives in Asia-Pacific Region [7]. The efforts to control dengue vectors mainly rely on the use of insecticides and reducing the breeding habitats, nevertheless dengue vectors are regularly invading into the new areas. Dengue vectors primarily bite during the day time therefore protection methods such as use of LLINs may not be equally effective against these vectors. Furthermore, widespread prevalence of resistance against the commonly used larvicides and application of ineffective methods or ineffective delivery of control methods could undermine the interventions that intend to reduce dengue vectors abundance [4, 8]. Hence efforts aiming at dengue vectors control have not been able to significantly reduce the dengue fever incidences in endemic countries. Consequently, strategies including the use of alternative options that could complement the existing intervention efforts need to be developed and employed in the vector mosquito control.

Pyriproxyfen (4-phenoxyphenyl (RS)-2-(2-pyridyloxy) propyl ether) is a pyridine based juvenile hormone analogue that acts as insect growth regulator (IGR). Among insects, the hormonal activity interferes in the normal process of embryogenesis and metamorphosis to inhibit many important processes including reproduction and development. The developmental changes in insects take place through moulting, which continues through the egg, larvae and pupae stages and before ending in the adult stage. Pyriproxyfen (PPF) is mainly involved in inhibiting the emergence of adult from pupal stage, however it has also been found associated in interfering different developmental stages in the insects [7–11]. Some recent studies have shown that use of PPF against dengue vector larvae efficiently reduced the dengue incidences in the intervention population [12]. Unlike the insecticides that are commonly used to kill the insects, PPF is not recognized as an adulticide and generally used in mosquito breeding sites for the control of mosquito vectors [11, 13]. Nevertheless, many studies have highlighted its role in reducing the longevity of the exposed insects [14–16]. Ohashi et al. [14] has shown that PPF treated net exposure decreased the survival rate of exposed *Anopheles* mosquitoes. This study further mentions that PPF exposed mosquitoes died within 8 days after exposure.

Nevertheless PPF is not well appreciated as mosquito adulticide, yet many studies have demonstrated that PPF elicit delayed residual effect that impairs the insect reproductive capacity by affecting the fecundity and fertility of adult mosquitoes exposed through tarsal contact. This effect may depend on the dose and time of exposure with respect to the blood meal. Ohba et al. [17] showed that exposure of female *Ae*. *albopictus* to bed net treated with 350 mg/m^2^ and 35 mg/m^2^ PPF effectively reduced the fecundity and egg hatchability. The study further suggested the auto-dissemination of PPF into breeding sites by adult mosquitoes through contact with PPF treated bed nets [17]. In *An*. *gambiae* and *Culex quinquefasciatus* adult females exposed to 2.6 mg/m^2^ PPF caused significant sterilizing effect between 24 hr before and after blood meal [18]. Koama et al. [19] studied that adult females *An*. *gambiae* exposed to 1% treated PPF net before and after blood meal resulted in nearly complete inhibition of fecundity and fertility. Furthermore, PPF has been found to be effective against the mosquitoes that have developed resistance to various insecticides [14, 20–23]. Organophosphate (OP) resistant strain of *Cx*. *quinquefasciatus* exposed to PPF in insecticide paint Inesfly 5A IGR caused reduction in fecundity, fertility and adult emergence [24]. PPF is safe to the non-target organisms and categorized in WHO list of chemicals that are unlikely to produce hazardous effect in normal use [25].

Present study was undertaken to evaluate the impact of PPF adsorbed surface exposure on Indian wild type *Ae*. *aegypti* at different concentrations to determine the minimum concentration of PPF on surface that could be effective in mosquito control.

## Material and methods

### Mosquitoes

Indian wild type *Ae*. *aegypti* larvae were collected from natural dwelling locations in field from Gwalior, Madhya Pradesh, India and maintained in the insectary of Vector Management Division, Defence Research and Development Establishment, Gwalior, India. Larvae were kept in disposable cups (100 ml capacity) in gauge wooden cages (size: 750mmX600mmX600mm) and given commercially available dog biscuits and yeast granules as food. The mosquito culture was maintained at 27±2°C temperature, 75±5% relative humidity (RH) and 12:12 [light:dark] hr. Cotton soaked with 10% sugar solution was provided *ad-libitum* to the adults for nourishment and the female mosquitoes were fed on rabbits for blood meal. Females of F1 population of the collected *Ae*. *aegypti* mosquitoes were used in the present study.

### Preparation of PPF treated papers and mosquito exposure

Four different concentrations of PPF (0.75%, 0.075%, 0.0075%, and 0.00075%) were prepared by diluting PPF (98.2% purity) in HPLC grade acetonitrile (Merck, India) and treatment on paper was done following standard method. The amount of PPF in 0.75% concentration was 4.95 mg which gives 275 mg/m^2^ concentration of active ingredient. The papers after treatment were left to air dry for 30 min at room temperature. Control papers were prepared using acetonitrile only. The impregnated papers used in the present study were prepared using 12 × 15 cms Whatman No. 1 filter papers (thickness—180 μm; pore size—11 μm). The experiments were carried out in batches of 5–7 females for each treatment (N ≥ 20). Fresh impregnated paper was used for every batch in each concentration. Female mosquitoes (4–5 days old) were exposed to impregnated and control papers in WHO bioassay test kits for 30 min and thereafter transferred to holding tubes. One cohort of mosquitoes was exposed to different concentrations 24 hr before blood meal, whereas a separate cohort was exposed 24 hr after the blood meal. Once both exposure and blood feed were complete, the females were maintained in PPF free cages containing non-chlorinated tap water filled cups lined with filter papers for oviposition. Total eggs laid by each batch in subsequent five days were recorded. The eggs that failed to hatch after seven days were treated as dead [26]. The effect of exposure was observed in terms of fecundity, fertility, pupae formation and adult emergence.

### Estimation of PPF by HPLC

Isocratic elution was performed by a mixture of HPLC grade acetonitrile (Merck, India) and water (Elix-MiliQ; filtered through 0.22 μM membrane filter) as mobile phase at 80:20 (v/v) (Waters 1525 binary HPLC pump; flow rate of 1 ml/min). Chromatographic separation was performed and signals were recorded at 280 nm (Waters XTerra C18 column 4.6 x 250 mm; 5μm; 2487 UV detector). Different concentration solutions of PPF (stock as well as working standards) were prepared in acetonitrile and injected through a Rheodyne injector fitted with 10 μL loop.

The PPF in impregnated papers was extracted in acetonitrile and estimated. Briefly, each paper (either impregnated or control) of area 180 cm^2^ was cut into four equal parts (45 cm^2^) and thereafter each part was cut into small pieces and extracted with acetonitrile (2 x 5 ml) in a 25 ml conical flask under sonication for 5 min. Resulting solution containing PPF was transferred to a 10 ml volumetric flask followed by volume make up with acetonitrile. Three samples were prepared for each part of paper. Each sample was used for HPLC and PPF content was calculated by using following equation:

$${\left[PPF\right]}_{sample}=4\;*\;P*{\left[PPF\right]}_{standard}\;*\;(A_{sample}/A_{standard})$$

Where [PPF] is concentration of PPF in mg/ml; A_standard_ and A_sample_ are peak areas of PPF in standard and sample solution respectively; P is the purity of standard PPF. Multiplication factor 4 was used because a quarter of each paper was used for sample preparation.

### Fertility and fecundity assessment

Average number of eggs laid by single female was determined for control as well as for all the concentrations. Inhibition (%) in fecundity was calculated as reduction in number of eggs laid per female for a given concentration relative to the control using following calculation;

%inhibitioninfecundity:100−[(Lt/Lc)x100]

where Lc is average number of eggs laid per female in the control, while Lt in a given treatment.

Similarly, average number of eggs hatched per female in control and treatment was used to determine the fertility inhibition, as follows;

%inhibitioninfertility:100−[(Ht/Hc)x100]

where Hc and Ht are average number of eggs hatched per female in the control and a given treatment respectively.

Furthermore, the percentage reduction in overall adult emergence per female in treatment relative to the control was determined using following equation;

%inhibitioninemergence:100−[(Et/Ec)x100]

where Ec and Et are the reproductive rates represented by the average number of adult emerged per female in control and a given treatment respectively.

### Ovary development

Developmental stages of ovaries in exposed as well as control female *Ae*. *aegypti* were observed after dissecting the mosquitoes used in the study. Altogether, 20 females from control, while 30 females from each exposure concentration were dissected under microscope (Leica, EZ4). Females were anesthetized at −20°C for 8–10 min and individually dissected in a drop of distilled water by gently pulling out the last abdominal segment. The extracted ovaries were studied under different magnifications of microscope (Leica, MZ 205A).

### Statistical analysis

Mean outcomes such as eggs laid, egg hatched, pupae formed and offspring emerged for control and treatments were compared using analysis of variance (ANOVA) followed by Tukey Kramer test of multiple comparison. Student’s ‘t’ test was used for comparison between the control and a given treatment. Percentage inhibition outcomes were subjected to probit analysis using log-probit method (Ldp Line software, Ehabsoft, Egypt) to find inhibitory concentrations for egg laid, egg hatched and offspring emerged. Chi square (χ^2^) test was used to assess the fitment of probit. The overall significance has been assessed using Bonferroni procedure. Concentration of PPF extracted from impregnated papers was presented as mean ± standard deviation.

### Ethical statement

This study was approved by the institutional animal ethical committee (IAEC) of DRDE, Gwalior, India vide research protocol number VMD-06/56/DS-I. The IAEC adheres to the guidelines of Committee for the Purpose of Control and Supervision of Experiments on Animals (CPCSEA), Animal Welfare Division, Government of India.

## Results

### Reduction in fecundity, fertility and emergence in female *Ae*. *aegypti* exposed 24 hr before blood meal

Indian wild type *Ae*. *aegypti* exposed to different concentrations of PPF impregnated on paper surface displayed considerable reproductive disruption (Tables 1 & 2). Average eggs laid, eggs hatched, pupae formed and adult emerged per female exposed before blood meal showed declining trend (S1 Fig) from lowest to highest concentration of PPF (F ≥ 41.9; p < 0.0001; R^2^ ≥ 0.94; df = 4). The control females not exposed to PPF from before blood meal group laid 64.1±1.5 (average ± standard error of mean) number of eggs, which was significantly higher as compared to all the test concentrations (t ≥ 8.9; p ≤ 0.01) (Table 1). Similarly average number of eggs hatched (fertility) and adult emerged per female was also high in control as compare to different concentrations of PPF used in the present study (t ≥ 10.5; p ≤ 0.009 for fertility and t ≥ 11.1; p ≤ 0.008 for adult emergence). Percentage inhibition in fecundity (egg laid) ranged from 36.9% [OR = 0.41 (95% CI = 0.24–0.71)] in 0.00075% to 45.1% [OR = 0.30 (95% CI = 0.17–0.54)] in 0.75% PPF concentration. Similarly % inhibition in fertility ranged from 46.9% [OR = 0.28 (95% CI = 0.16–0.51)] in the lowest concentration to 61.8% [OR = 0.15 (95% CI = 0.08–0.29)] in the highest concentration respectively. Furthermore, the inhibition (%) observed in adult emergence in females exposed to PPF before blood meal was found to be 58.8% [OR = 0.18 (95% CI = 0.09–0.36)] and 79.2% [OR = 0.04 (95% CI = 0.02–0.10)] in 0.00075% (lowest concentration) and 0.75% (highest concentration) PPF respectively. Probit analysis suggested that FI_50_ (50% fertility inhibition) value was 0.002% (p = 0.82), whereas EI_50_ (50% emergence inhibition) was 0.0001% (p = 0.99) respectively (Table 3). Presently the log-dose probit model used to determine the EI_50_ and FI_50_ values displayed normal distribution of percent EI and FI with concentration.

**Table 1:** Effect on *Ae*. *aegypti* fecundity, fertility and adult emergence when exposed before blood meal.

**Effect on fecundity of *Ae*. *aegypti* females exposed 24 hr before blood meal**					
**Exposure**	**Eggs laid** **(average** ±**SEM)**	**95% CI**	**Inhibition (%)**	**OR (95%CI)**	**p(t)**
Control	64.1±1.5	57.8–70.4	0	ND	ND
0.00075	40.5±1.6	33.5–47.4	36.9	0.41(0.24–0.71)	0.012(8.9)
0.0075	38.8±1.1	34.2–43.3	39.5	0.37(0.21–0.65)	0.002(23.6)
0.075	37.3±3.0	24.3–50.2	41.9	0.33(0.19–0.59)	0.004(16.3)
0.75	35.2±1.4	29.5–41.1	45.1	0.30(0.17–0.54)	<0.0001(257.6)
**Effect on fertility of *Ae*. *aegypti* females exposed 24 hr before blood meal**					
**Exposure**	**Eggs hatched (average ±SEM)**	**95% CI**	**Inhibition (%)**	**OR (95%CI)**	**p(t)**
Control	61.7±1.7	54.5–68.8	0	ND	ND
0.00075	32.8±1.5	26.2–39.3	46.9	0.28(0.16–0.51)	0.009(10.5)
0.0075	27.3±0.8	23.7–30.9	55.7	0.19(0.10–0.36)	0.002(25.5)
0.075	26.4±2.1	17.6–35.3	57.1	0.18(0.09–0.34)	0.0002(69.9)
0.75	23.6±1.5	17.3–29.8	61.8	0.15(0.08–0.29)	0.0003(54.0)
**Effect on adult emergence of *Ae*. *aegypti* females exposed 24 hr before blood meal**					
**Exposure**	**Emergence** **(average** ±**SEM)**	**95% CI**	**Inhibition (%)**	**OR (95%CI)**	**p(t)**
Control	50.1±2.5	39.5–60.8	0	ND	ND
0.00075	20.7±0.7	17.7–23.6	58.8	0.18 (0.09–0.36)	0.008(11.1)
0.0075	16.6±0.2	15.7–17.4	67.0	0.12 (0.05–0.25)	0.005(13.7)
0.075	13.5±1.3	8.0–19.1	73.0	0.08 (0.03–0.18)	0.001(30.2)
0.75	10.4±0.4	8.5–12.3	79.2	0.04 (0.02–0.10)	0.002(19.3)

ND- not determined; CI-confidence interval; OR-odds ratio; SEM-standard error of mean.

**Table 2:** Effect on *Ae*. *aegypti* fecundity, fertility and adult emergence when exposed after blood meal.

**Effect on fecundity of *Ae*. *aegypti* females exposed 24 hr after blood meal**					
**Exposure**	**Eggs laid** **(average** ±**SEM)**	**95% CI**	**Inhibition (%)**	**OR (95%CI)**	**p(t)**
Control	65.1±6.0	39.4–90.7	0	ND	ND
0.00075	49.5±3.6	34.0–65.0	24.0	0.59(0.35–1.00)	0.02(6.6)
0.0075	45.1±0.2	44.1–46.2	30.7	0.48(0.28–0.82)	0.04(3.3)
0.075	42.8±5.7	18.5–67.1	34.3	0.44(0.25–0.75)	0.001(32.1)
0.75	44.3±0.8	41.1–47.5	32.0	0.46(0.27–0.79)	0.04(3.1)
**Effect on fertility of *Ae*. *aegypti* females exposed 24 hr after blood meal**					
**Exposure**	**Eggs hatched (average ±SEM)**	**95% CI**	**Inhibition (%)**	**OR (95%CI)**	**p(t)**
Control	55.8±5.1	33.7–77.7	0	ND	ND
0.00075	33.5±2.6	22.3–44.7	40.0	0.37(0.20–0.67)	0.01(8.8)
0.0075	27.7±0.2	26.9–28.4	50.4	0.25(0.13–0.47)	0.03(5.4)
0.075	25.1±1.6	18.1–32.2	55.0	0.20(0.10–0.39)	0.02(8.0)
0.75	25.5±0.4	23.9–27.0	54.3	0.22(0.11–0.42)	0.03(6.0)
**Effect on adult emergence of *Ae*. *aegypti* females exposed 24 hr after blood meal**					
**Exposure**	**Emergence (average ±SEM)**	**95% CI**	**Inhibition (%)**	**OR (95%CI)**	**p(t)**
Control	43.3±4.0	27.4–59.2	0	ND	ND
0.00075	15.4±1.2	10.4–20.3	64.4	0.12(0.05–0.28)	0.01(11.0)
0.0075	11.5±0.6	8.97–14.0	73.4	0.08(0.03–0.19)	0.02(7.6)
0.075	9.9±1.3	4.38–15.5	77.1	0.05(0.02–0.14)	0.01(13.7)
0.75	10.7±0.5	8.6–12.7	75.3	0.07(0.03–0.17)	0.01(8.8)

ND- not determined; CI-confidence interval; OR-odds ratio; SEM-standard error of mean.

### Reproductive disruption impact on female *Ae*. *aegypti* exposed 24 hr after blood meal

Impact of PPF exposure on reproduction competence of female *Ae*. *aegypti* mosquitoes has been presented in Table 2. There was significant concentration dependent change in fecundity, fertility and adult emergence as number of eggs laid, eggs hatched and adult emerged per female were found decreasing with increasing concentration (F ≥ 5.2; p < 0.02; R^2^ ≥ 0.67; df = 4). It was found that average eggs laid, hatched and adult emerged for placebo control females were statistically more than females exposed to different concentrations (t ≥ 5.4; p ≤ 0.03) (Table 2 & S2 Fig). Results showed that fecundity inhibition (%) ranged from 24.0% [OR = 0.59 (95% CI = 0.35–1.00)] to 34.3% [OR = 0.44 (95% CI = 0.25–0.75)]. On the other hand, inhibition in fertility was found ranging from 40.0% [OR = 0.37 (95% CI = 0.20–0.67)] in 0.00075% to 55.0% [OR = 0.20 (95% CI = 0.10–0.39)] in 0.075% of PPF concentration respectively. Similarly, the adult *Ae*. *aegypti* emergence inhibition varied from 64.4% [OR = 0.12 (95% CI = 0.05–0.28)] to 77.1% [OR = 0.05 (95% CI = 0.02–0.14)] among the different concentrations used in the present study (Table 2). In females exposed to PPF 24 hr after blood meal, the FI_50_ value was 0.01% (p = 0.63), whereas EI_50_ was observed to be <0.0001% (p = 0.98) respectively (Table 3). Furthermore, the log dose probit model used to determine FI_50_ and EI_50_ did not deviate from the linearity (p≥0.05) (Table 3).

**Table 3:** Probit analysis of fertility and adult emergence inhibition impact of PPF exposure on *Ae*. *aegypti* mosquitoes.

Exposure	Effect	Concentration (%)	χ^2^(p)	Slope	r^2^	g
Before blood meal	FI_50_	0.002	0.4(0.82)	0.12±0.06	0.96	0.90
	EI_50_	0.0001	0.02(0.99)	0.20±0.06	1.0	0.36
After blood meal	FI_50_	0.01	0.2(0.63)	0.19±0.09	0.98	0.86
	EI_50_	<0.0001	0.02(0.98)	0.19±0.09	0.98	0.98

FI_50_/EI_50_-fertility/adult emergence inhibition 50%; χ2 –chi square

Results have also suggested that reduction found in egg laying, egg hatching and pupae formation was similar in both the exposure groups (24 hr before blood meal group and 24 hr after blood meal group), but differ statistically in adult emergence for all the concentrations (p≤ 0.02; t≤ 3.9), except for 0.75% (p = 0.8; t = 0.3).

### Impact on ovary maturation

**Fig 1:**
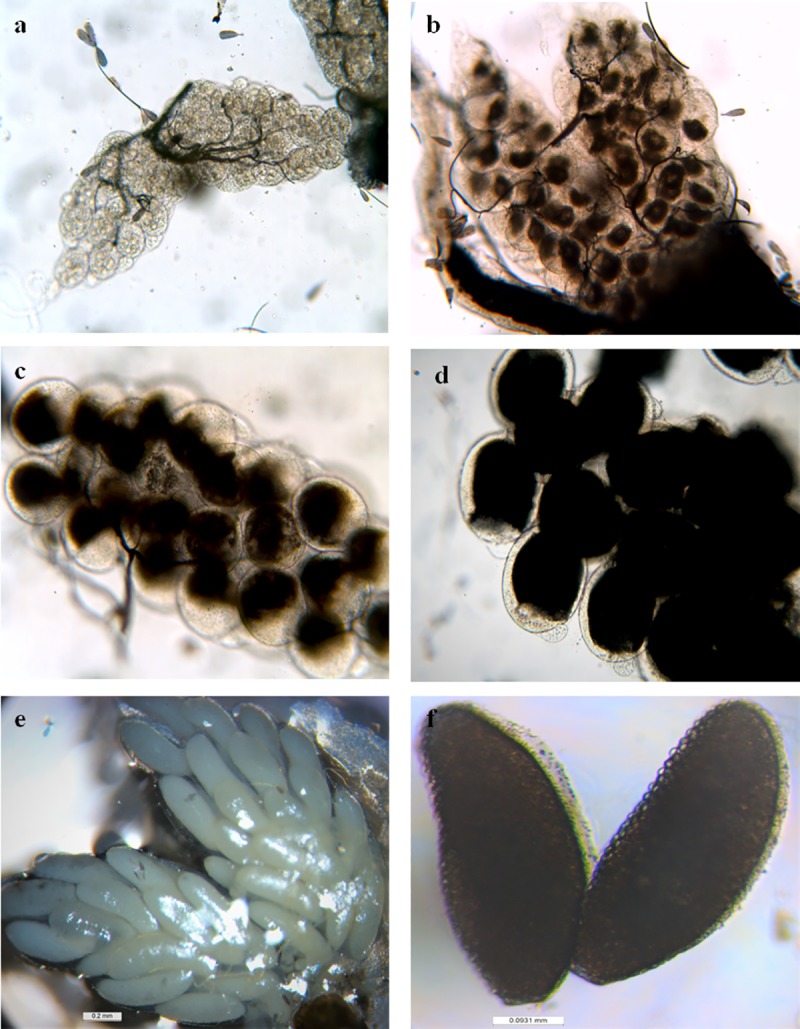
Sequential development stages of ovaries in control *Ae*. *aegypti* female. (a) Before blood meal, (b) after 18 hr of blood meal, (c) after 24 hr of blood meal, (d) after 48 hr of blood meal, (e) after 72 hr of blood meal, (f) fully mature single egg seen after 72 hr of blood meal.

**Fig 2:**
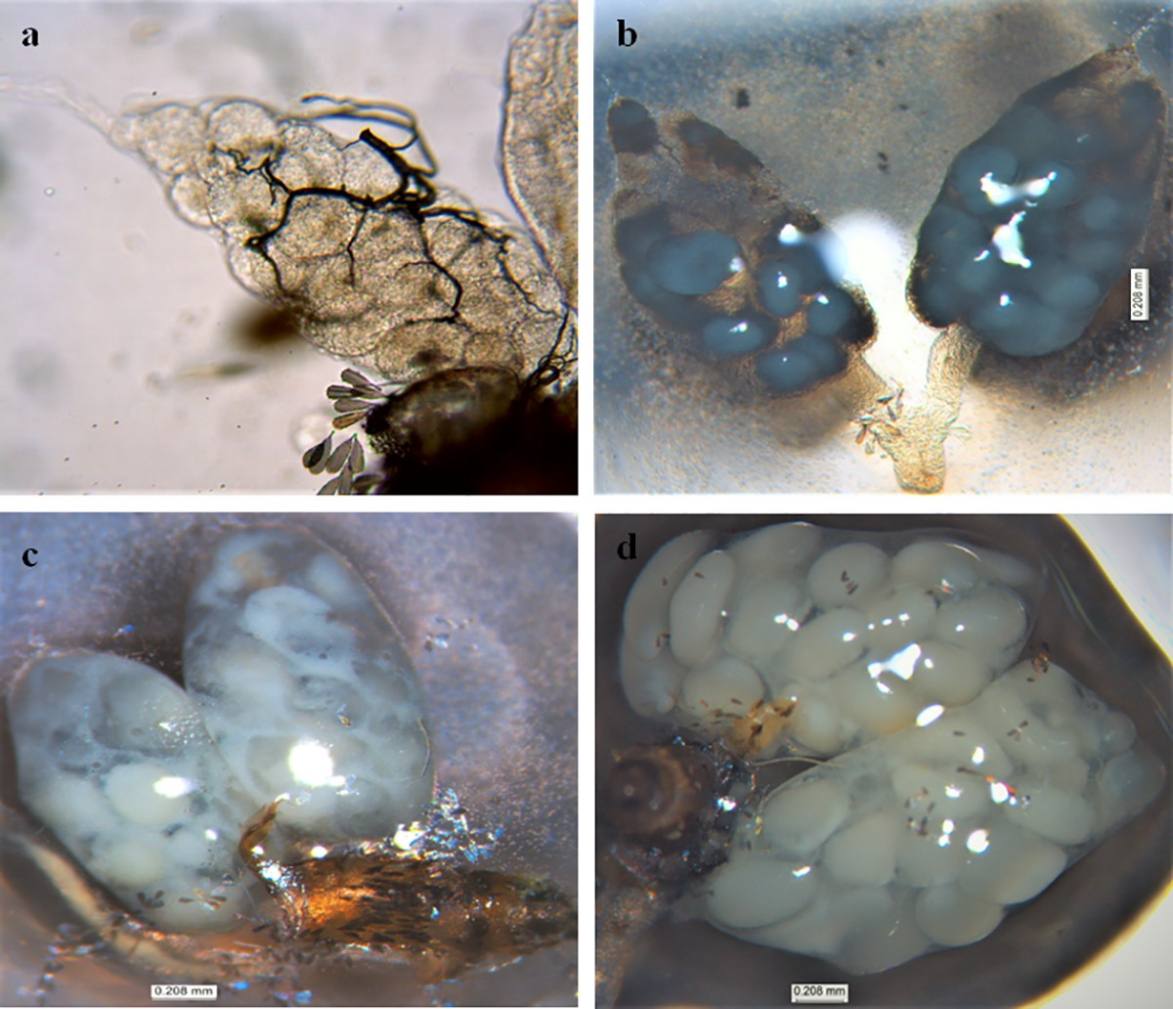
Ovary of exposed female *Ae*. *aegypti*. (a) Eggs showing arrested yolk formation, (b) ovarioles showing distorted eggs formation, (c) showing abnormal aborted egg (d) showing retarded growth in egg development.

**Fig 3:**
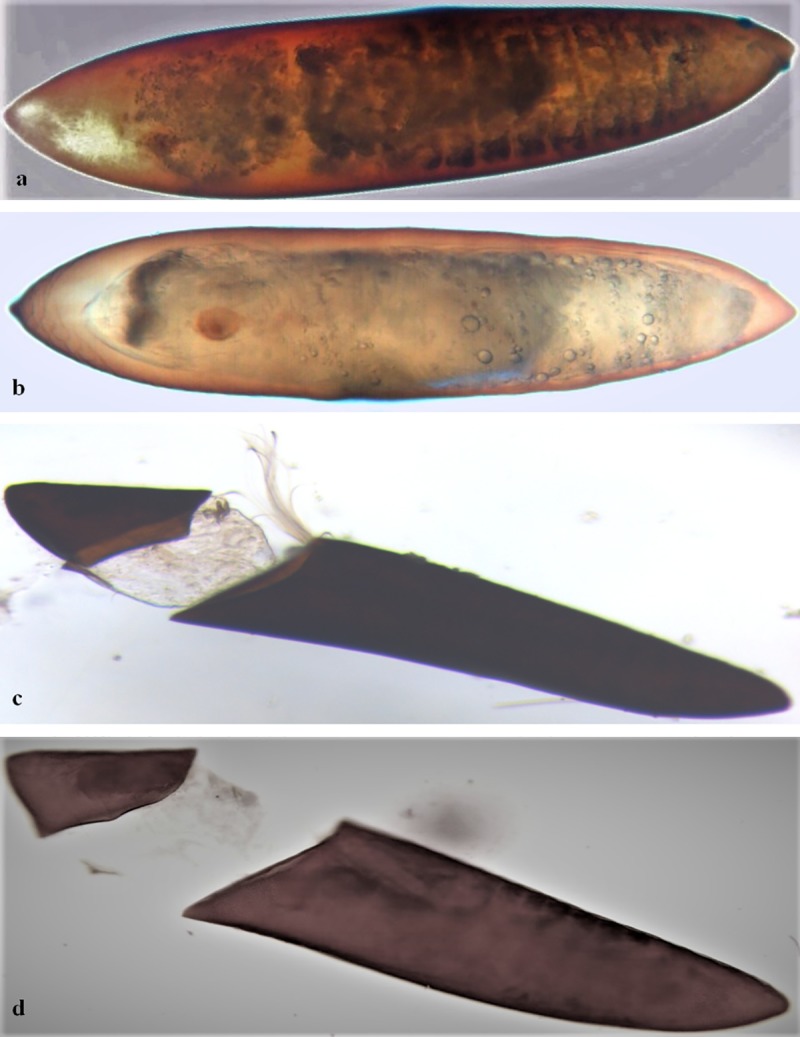
Eggs laid by female *Ae*. *aegypti*. (a) Control egg showing normal segmentation, (b) egg of exposed female displaying disrupted differentiation and no segmentation, (c and d) abnormal hatching of eggs of exposed female showing death of larval stage.

We could not find any visible difference between ovaries of control group females and those exposed at concentrations lower than 0.75%. In control group, the ovaries displayed normal vitellogenesis during 18 hr, 24 hr and 48 hr, and fully developed eggs post 72 hr of blood meal (Fig 1a–1f). However in 0.75% exposure group, no vitellus formation could be seen after 18 hr of blood feed (Fig 2a), whereas after 72 hr there was arrest in follicle maturation as the eggs could not attain Christopher stage V (Fig 2b–2d). Of this group, only 11 (36.7%) females showed microscopically distinguishable morphological deformities as compared to the control. Furthermore, in successfully laid eggs of exposure group females, there was altered body organization, chitinization and disoriented cervical and abdominal region as compared to control (Fig 3a and 3b). Such eggs either did not hatch or hatched improperly and died subsequently (Fig 3c and 3d).

### Concentration and uniformity of PPF on impregnated papers

Presently, the isocratic method of elution used for quantitative analysis of PPF (S3 Fig) provided an excellent correlation coefficient (0.998) between peak area and concentration of standard PPF solutions in the working range of 0.00012375 to 0.12375 mg/ml (S1 Table & S4 Fig). At present, this method has been found effective in separating the PPF from other interfering chemicals extracted from the impregnated paper (S5 Fig). It was found that in 0.00075% concentration impregnated paper, HPLC method estimated 0.00067 ± 0.00011%, whereas for 0.75%, it estimated 0.748 ± 0.059% quantitatively (S2 Table). Results further showed that the impregnation of PPF was uniform throughout the paper. Nevertheless, in all cases, the HPLC estimated less PPF than impregnated initially on the paper and the degree of estimation ranged from 0.72–0.98.

## Discussion

Amidst reports of widespread insecticide resistance to different mosquito vectors, priority is to use chemicals that can improve control interventions and are safe to non-target organisms. In the recent years, PPF delivered using different methods has been shown to effectively control immature stages of vector mosquitoes in variety of habitats in endemic regions. The impact of PPF on aquatic stages of vector mosquitoes has been much studied primarily because control measures using PPF are mostly aimed at targeting developmental stages that are present in breeding habitat [10, 16, 26, 27]. However its efficacy and persistence in aquatic conditions has been inconsistent due to various reasons [16]. On the other hand its usefulness while treated on surfaces, such as nets, cloths, paints and spray formulations, which are used in human dwellings, could be more practical in achieving sustainable persistence and efficacy for a considerable time.

Current study has demonstrated that PPF impregnated on surface at very low concentration is capable of interrupting reproduction in field collected vector mosquitoes. *Ae*. *aegypti* females exposed to a range of PPF concentrations revealed reduction in egg laying, egg hatching and adult emergence. There was concentration dependent non-significant decline in egg laying in exposed female (both exposures), indicating that minimum concentration used in the study was equally capable in reducing the fecundity (p≥0.3). However in case of fertility and adult emergence reduction, there was concentration dependent, but significant effect as the impact was more in higher concentrations (p≤0.02; F≥6.2). This suggests that PPF present on a surface in lower concentration can reduce the egg laying ability among exposed female mosquitoes to the same extent as that of higher concentration. Overall the impact of PPF was found hampering egg laying, egg hatching and adult emergence when exposed to all the concentrations. The results have suggested that a concentration of 0.0001% PPF impregnated on surface could be sufficient to considerably reduce the adult emergence in dengue vector *Ae*. *aegypti*. In an earlier study, PPF treated on hard surface has been demonstrated to produce reduction in different developmental stages in *An*. *gambiae* and *Cx*. *quinquefasciatus* mosquitoes with a reduction ranging from 60 to 94% as compared to control [18]. Furthermore, the study also showed that emergence inhibition was high when females were exposed immediately before oviposition. In the present study, the reduction compared to control females never inclined above 79.2%, but the emergence inhibition impact was similar to that reported by Mbare et al [18]. Studies have also reported that higher concentrations used on hard surfaces are capable of producing complete sterility in exposed females. Koama et al. [19] has suggested that females of *An*. *gambiae* exposed to 1% PPF treated bed nets could not lay eggs even after many blood meals, indicating that higher concentration of PPF could induce persistent and longer residual activity on mosquito reproduction. In contrast, another study [21] revealed that 1% PPF alone or in combination with permethrin could reduce fertility by 7–12% relative to control, while no effect could be found in fecundity of wild resistant *An*. *gambiae* s.s. in field experiments. Therefore it is more likely that overall impact of PPF may vary depending on concentration used, mode of delivery, exposure time, size of mosquito, and other abiotic factors [10, 11, 16].

Several studies have suggested that effectiveness of PPF decreases over time as the concentration present on the treated surface and that available to the exposed insects decline with time [9, 16]. Keeping this in mind, present study has used different concentrations offering wide range of dose exposure to the tested female mosquitoes to understand whether reduced concentration expected over a period of time could sufficiently impact the mosquito development. Present results have indicated that a concentration of 0.002% and 0.0001% were enough to reduce fertility and adult emergence respectively by 50% in females exposed before blood meal. Similarly, 0.01% and <0.0001% concentrations could be able to cause 50% reduction in fertility and adult emergence in females exposed after blood meal.

Exposure to PPF-treated surface prevented the rate at which *Ae*. *aegypti* female ovaries develop through different previtellogenic stages to attain maturity. It has been reported that PPF based formulations obstruct the previtellogenic developmental process and block the yolk proteins synthesis, hence disrupting the hormonal route necessary for egg development [19]. Microscopic observation of control female ovaries showed that ovarioles followed a sequential developmental pattern showing vitellus agglomeration at the base of follicles and contained a single large oocyte as compared to the exposed ovarioles where oocytes were round and much reduced in size (Fig 1). The exposed females showed deterioration of follicular membrane and cytoplasm in the ovarioles, and reduced vitellus granules in the follicles during the initial 18 hr of blood meal (Fig 2a). In some cases, ovaries contained a few ovarioles only that were dark in colour (Fig 2b), more opaque and sometime with diffused and improperly organized eggs (Fig 2c and 2d), hence displaying clear signs of abortion. In Africa, the females of malaria vector *An*. *gambiae* exposed to PPF treated nets showed delayed development that never reached to maturity [19]. Furthermore, PPF treatment has been found associated with the abnormal development of eggs in different vector mosquito species by arresting the embryo development at different development stages [26]. There was explicit segmentation showing clear body division in the eggs that were laid by control females (Fig 3a), whereas the exposed female eggs showed distinct desegmentation in the eggs (Fig 3b). In some cases, the eggs in exposed females could not hatch properly and died subsequently. Such eggs showed undifferentiated mass of underdeveloped larvae attached to operculum as well as to the larger abopercular portion of the egg (Fig 3c and 3d). Suman et al. [26] has reported that some IGR analogues are capable of causing morphological deformities and disoriented body organisation, however they showed that PPF alter the hormonal actions during embryogenesis but may not affect the egg hatching process.

Cross estimation of PPF on impregnated papers using HPLC method was carried out to find out the concentration of free PPF available for exposed mosquitoes. Various studies have argued that chromatographic methods could be used to estimate the decline in insecticide residue over a period of time [28], but it is equally important to first estimate the initial concentration of active anti-insect chemical and then determine the decline in light of estimated concentration. Presently used HPLC method was effective to extract and estimate PPF, and displayed >0.72 degree of estimation as compared to a previous study that could estimate 60.9% of permethrin [29]. Due to excellent correlation between concentration and peak area, this method can suitably be employed to estimate the concentration and to check the uniformity of PPF in impregnated paper. The small variation in estimated PPF concentrations to treated concentrations may be due to various factors associated with impregnation process and the inherent property of the paper to hold back PPF tightly in its molecular architecture. Therefore, despite higher PPF concentration present in the treated matrix, the bioactivity depends upon the actual concentration available on the surface.

Presently, majority of the studies have demonstrated that PPF could be used effectively when applied against larval stages of vector mosquitoes, while few have suggested that PPF impregnated on nets was able to reduce the vector population in different areas. However data on efficacy of similar formulations that could be applied on surfaces such as walls, ceilings and other similar objects is scanty. Furthermore, none of the study to our knowledge has demonstrated the minimum concentration of PPF present in any surface/matrix that would be capable of reducing the mosquito population while delivered through tarsal contact. Present results provided sufficient evidence that PPF exposure caused irreversible structural damage to the ovaries of female *Ae*. *aegypti* mosquitoes, thereby making them unfit to lay normal eggs under laboratory condition. The PPF based formulations applied on different surfaces could be equally effective even after the concentration is reduced to minimum. These minimum effective concentrations can be used as cut-off limits for determining effective life of similar formulations that use PPF alone or in combination with the insecticides against mosquito vectors of public health importance. It might be possible for exposed female mosquitoes to carry PPF into the nearby breeding habitats. Since very small concentration of PPF is sufficient enough, transferring pyriproxyfen into small and cryptic mosquito breeding habitats through adults as vehicles could be an added advantage. PPF could be perfect to be used along with suitable insecticide for better control interventions in the areas where insecticide susceptibility has been compromised. Although, PPF used in formulations that offer its delivery through contact provides a promising strategy for reducing *Aedes* abundance, its similar effectiveness on other vector mosquitoes and role in reduction of disease incidences need to be established in field for better results in control programmes.

### Conclusion

PPF impregnated on surface substantially reduced the fecundity, fertility and adult emergence in Indian wild *Ae*. *aegypti* mosquitoes. Although the study did not investigate the relation between intrinsic activity of PPF in light of the behaviour of vector mosquitoes, but suggested that a certain minimum concentration of PPF through tarsal contact can reduce the vector mosquitoes population to a considerable level. The study advocates that formulations based on combination of PPF and other compatible insecticides may be an impactful approach where susceptible mosquitoes are killed by insecticide component and remaining resistant mosquitoes are sterilised by PPF. More importantly, integrating PPF in insecticide based vector control formulations that are used indoors could be more promising and control oriented. The results of current study can also be utilised to ascertain the effectiveness of PPF based formulations applied on surface by quantitative analysis rather performing time consuming and laborious bioassay experiments.

## Supporting information

S1 Fig Reproductive impact of PPF concentrations on *Ae. aegypti* exposed before blood meal. (a) egg laying, (b) eggs hatching, (c) pupation and (d) adult emergence [values: median (min to max) per female].
(TIF)

S2 Fig Reproductive impact of PPF concentrations on *Ae. aegypti* exposed after blood meal. (a) egg laying, (b) eggs hatching, (c) pupation and (d) adult emergence [values: median (min to max) per female].
(TIF)

S3 Fig Standard HPLC chromatogram of PPF. HPLC chromatogram of standard PPF solution (Rt = 5.993 min) at 0.001238 mg/ml concentration.
(TIF)

S4 Fig PPF concentration vs peak area. Correlation between concentration (mg/ml) and corresponding peak area of PPF in HPLC analysis in the working range of 0.00012375 to 0.12375 mg/ml concentration.
(TIF)

S5 Fig HPLC chromatogram of extracted PPF. Representative HPLC chromatogram of PPF solution extracted from impregnated paper. Peak at 5.999 min corresponds to PPF.
(TIF)

S1 Table Concentration of PPF (mg/ml) and corresponding peak area at 280 nm. (DOCX)

S2 Table Actual and estimated concentrations of PPF in impregnated papers used in the study. (DOCX)
