# Supplementary figures and images for: Pyriproxyfen treated surface exposure exhibits reproductive disruption in dengue vector Aedes aegypti
Source: PLoS Negl Trop Dis. 2019 Nov 18;13(11):e0007842. doi: 10.1371/journal.pntd.0007842 (PMC6886876; doi:10.1371/journal.pntd.0007842)

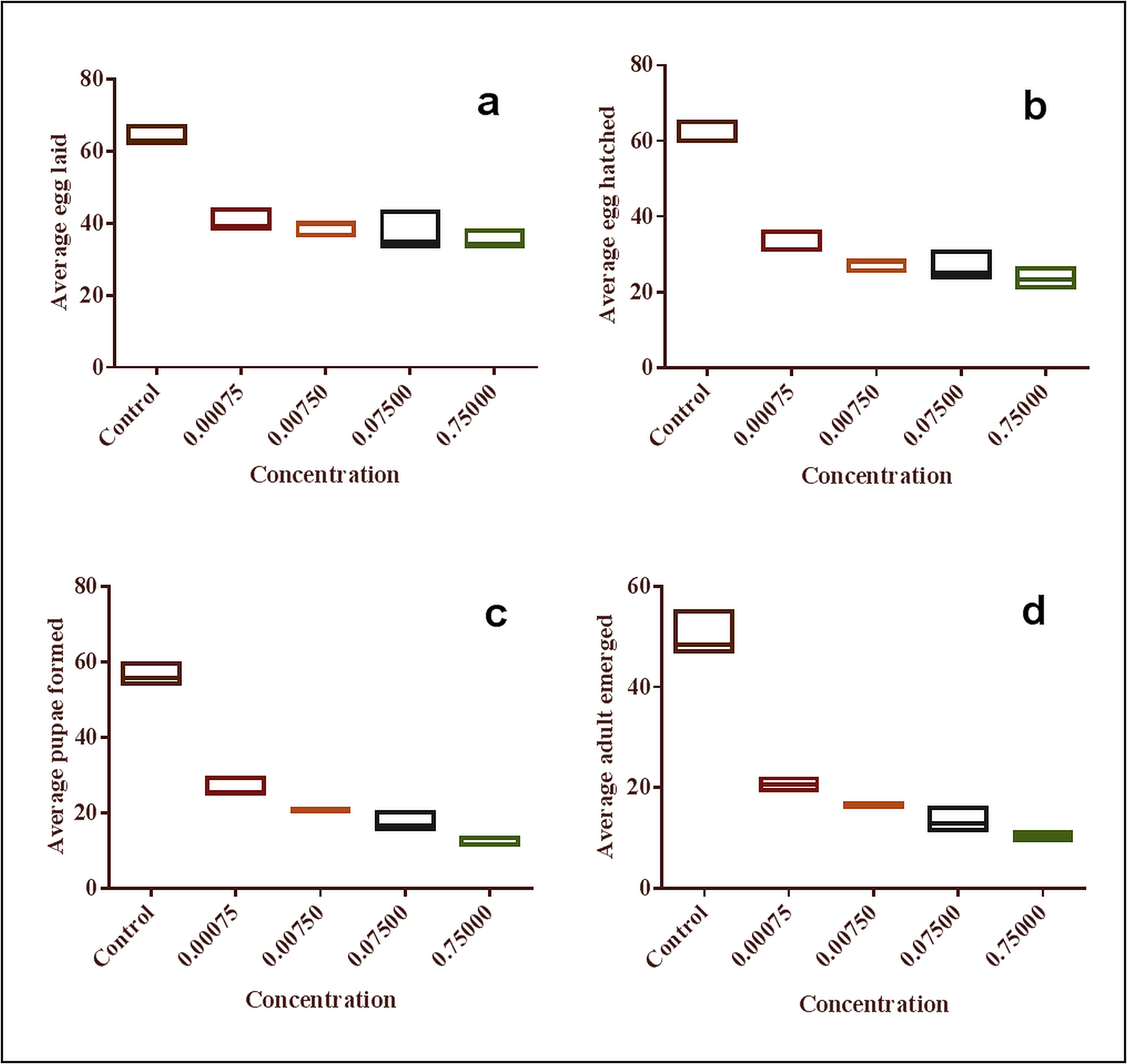

Supplement: S1 Fig — (a) egg laying, (b) eggs hatching, (c) pupation and (d) adult emergence [values: median (min to max) per female]. (TIF) [file pntd.0007842.s001.tif]

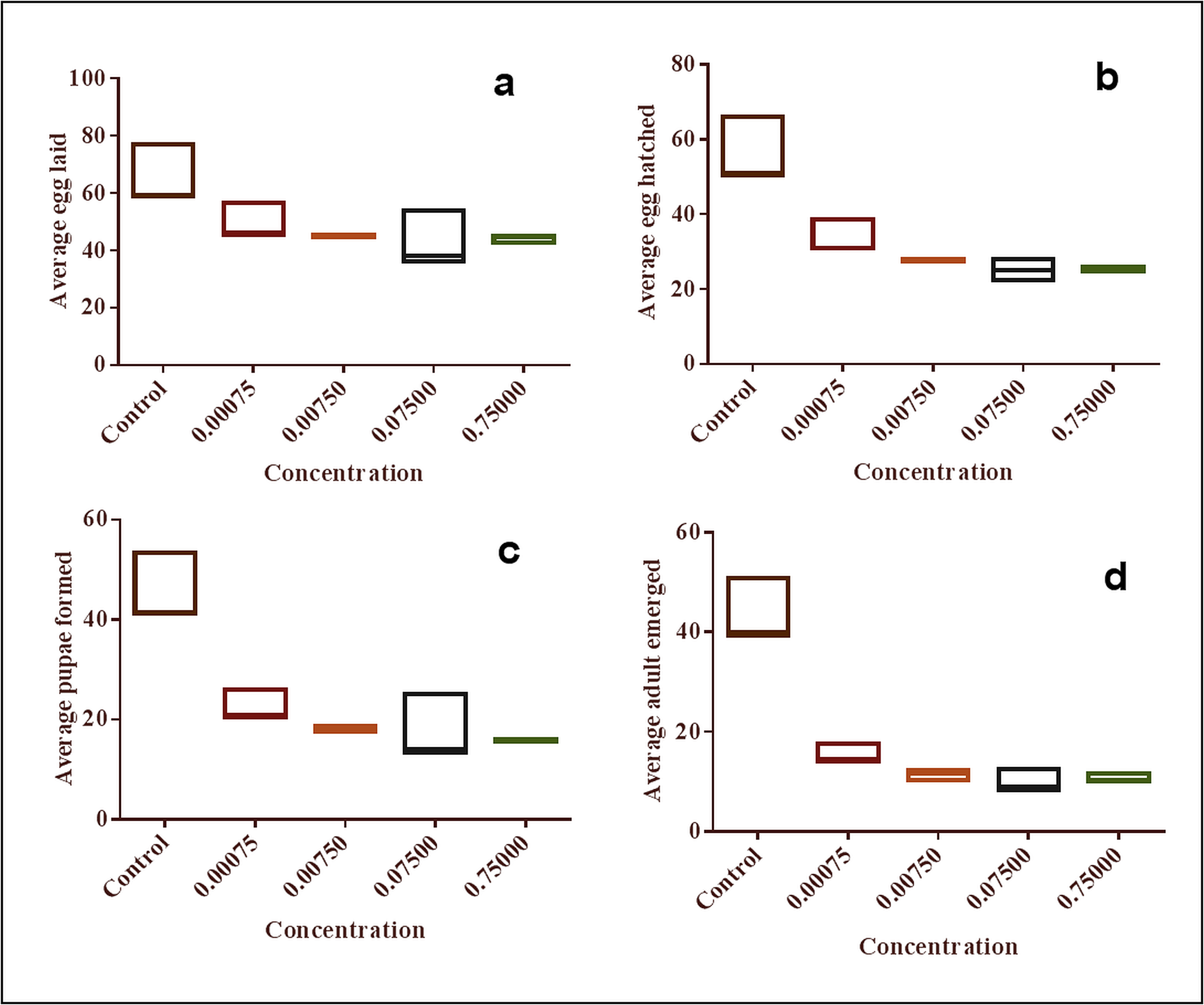

Supplement: S2 Fig — (a) egg laying, (b) eggs hatching, (c) pupation and (d) adult emergence [values: median (min to max) per female]. (TIF) [file pntd.0007842.s002.tif]

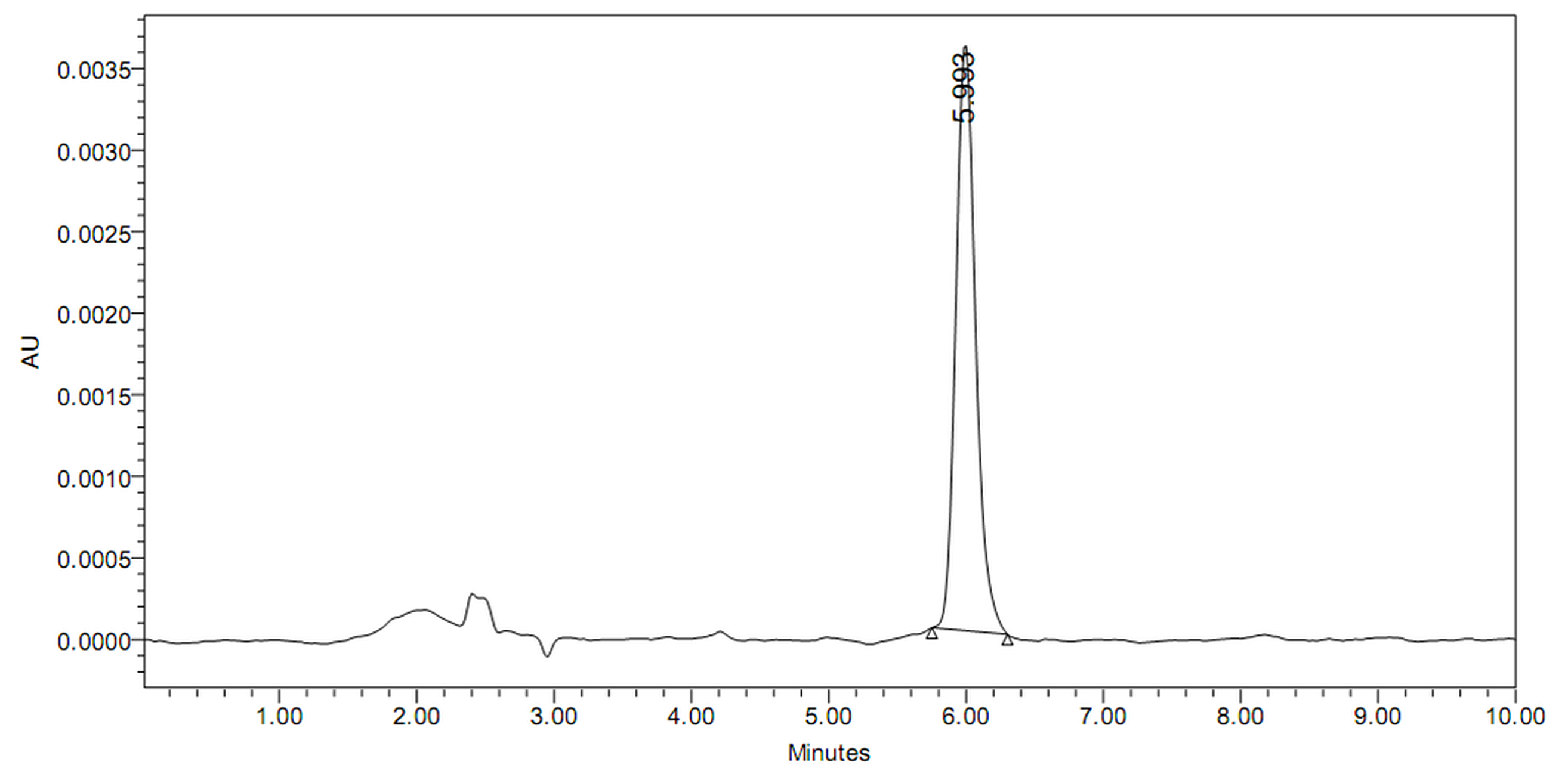

Supplement: S3 Fig — HPLC chromatogram of standard PPF solution (Rt = 5.993 min) at 0.001238 mg/ml concentration. (TIF) [file pntd.0007842.s003.tif]

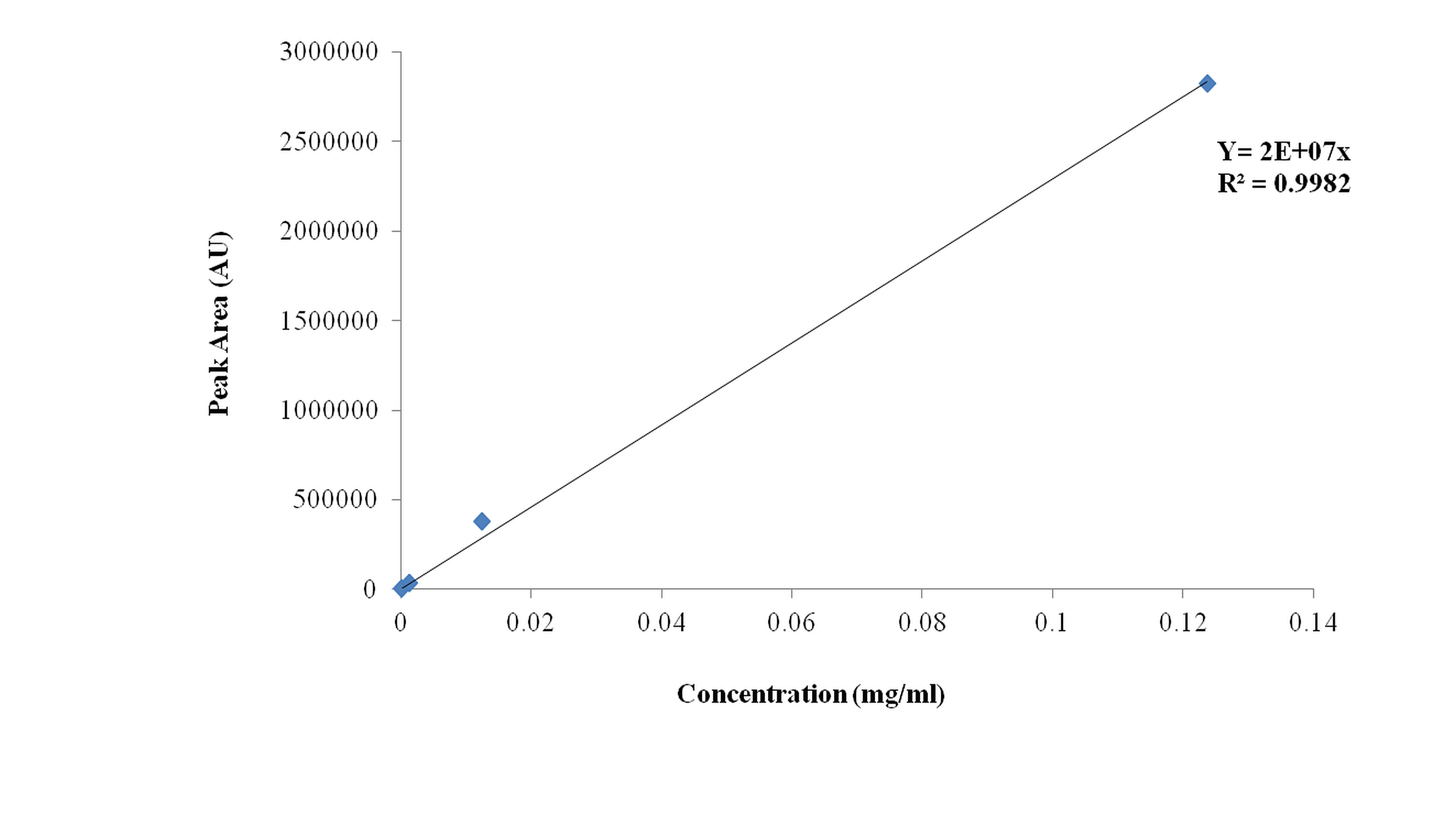

Supplement: S4 Fig — Correlation between concentration (mg/ml) and corresponding peak area of PPF in HPLC analysis in the working range of 0.00012375 to 0.12375 mg/ml concentration. (TIF) [file pntd.0007842.s004.tif]

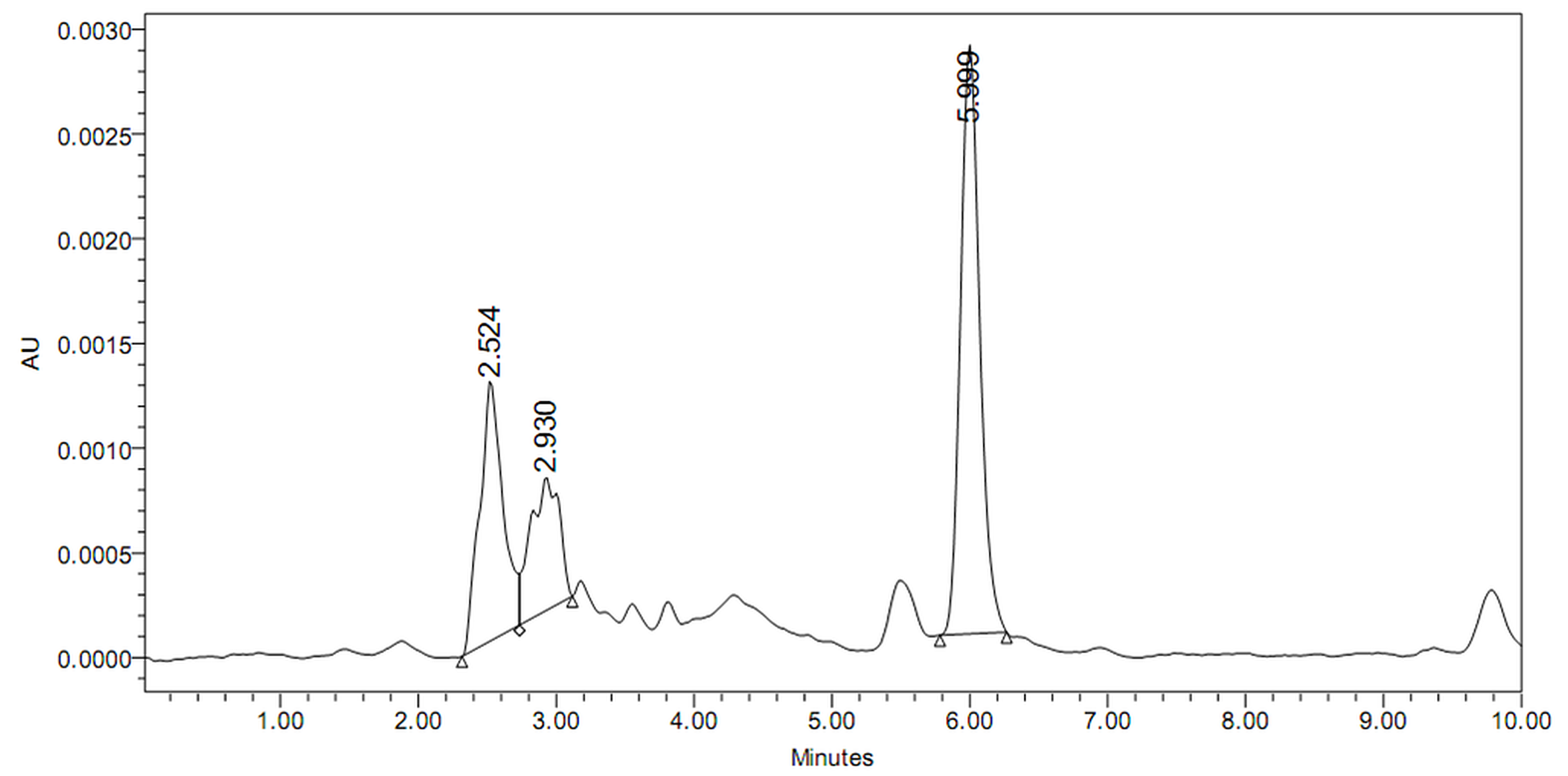

Supplement: S5 Fig — Representative HPLC chromatogram of PPF solution extracted from impregnated paper. Peak at 5.999 min corresponds to PPF. (TIF) [file pntd.0007842.s005.tif]
