# Supplementary material for: Pyriproxyfen treated surface exposure exhibits reproductive disruption in dengue vector Aedes aegypti
Source: PLoS Negl Trop Dis. 2019 Nov 18;13(11):e0007842. doi: 10.1371/journal.pntd.0007842 (PMC6886876; doi:10.1371/journal.pntd.0007842)
Supplement: S1 Table — (DOCX) [file pntd.0007842.s006.docx]

**Table 4: Concentration of PPF (mg/ml) and corresponding peak area at 280 nm.**

| **Concentration of PPF (mg/ml)** | **Peak area in arbitrary unit (AU)**  **(Average of two readings)** |
| --- | --- |
| 0.00012375 | 3432 |
| 0.0012375 | 35348 |
| 0.012375 | 382033 |
| 0.12375 | 2824440 |
