# Supplementary material for: Pyriproxyfen treated surface exposure exhibits reproductive disruption in dengue vector Aedes aegypti
Source: PLoS Negl Trop Dis. 2019 Nov 18;13(11):e0007842. doi: 10.1371/journal.pntd.0007842 (PMC6886876; doi:10.1371/journal.pntd.0007842)
Supplement: S2 Table — (DOCX) [file pntd.0007842.s007.docx]

**Table 5: Actual and estimated concentrations of PPF in impregnated papers used in the study.**

| **PPF concentration** | | | **HPLC estimate of PPF** | | **Degree of estimation** |
| --- | --- | --- | --- | --- | --- |
| **In paper (%)** | **In paper (mg/m^2^)** | **After extraction* (mg/ml)** | **after extraction***  **(mg/ml)** | **PPF concentration in paper (%)** |  |
| 0.00075 | 0.275 | 0.000495 | 0.00044± 0.00007 | 0.00067± 0.00011 | 0.89 |
| 0.0075 | 2.75 | 0.00495 | 0.00390± 0.00078 | 0.00592± 0.00118 | 0.79 |
| 0.075 | 27.5 | 0.0495 | 0.03567± 0.00495 | 0.054± 0.007 | 0.72 |
| 0.75 | 275 | 0.495 | 0.484± 0.038 | 0.748± 0.059 | 0.98 |

***** impregnated paper extracted with acetonitrile
